# Supplementary material for: Mechano-bactericidal activity of cicada wing nanostructures against gram-positive bacteria
Source: Microbiol Spectr. 2026 Jan 12;14(2):e02037-25. doi: 10.1128/spectrum.02037-25 (PMC12889022; doi:10.1128/spectrum.02037-25)
Supplement: Supplemental material — Fig. S1 and S2. [file spectrum.02037-25-s0001.docx]

Supplementary Information

Mechano-Bactericidal Activity of Cicada Wing Nanostructures Against Gram-Positive Bacteria

Jianwei Qu^1^, Shiya Gu^1^, Lei Chen^1^, Fangming Cui^1^, Huan Wang^1^, Liyan Wu^1*^

^1^ Shenyang Agricultural University, Shenyang 110866, China

^*^Authors to whom correspondence should be addressed.


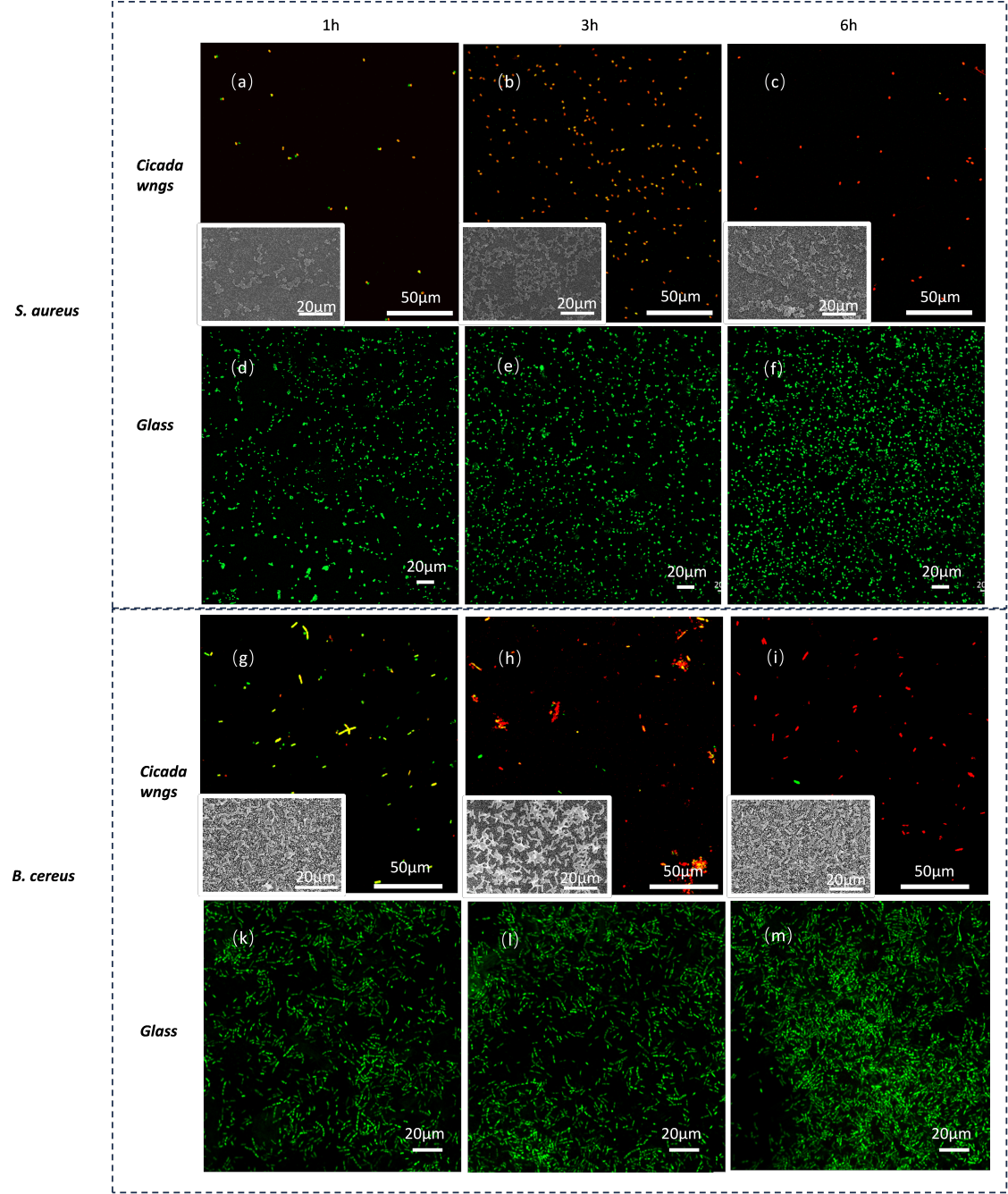


**Fig. S1.** CLSM and SEM images of bacterial attachment on the cicada wing surfaces. *S. aureus* on cicada wings (a and c) and glass (d and f); *B. cereus* on cicada wings (g and i) and glass (k and m). The lower-left insets show SEM images of bacterial adhesion on the respective surfaces.


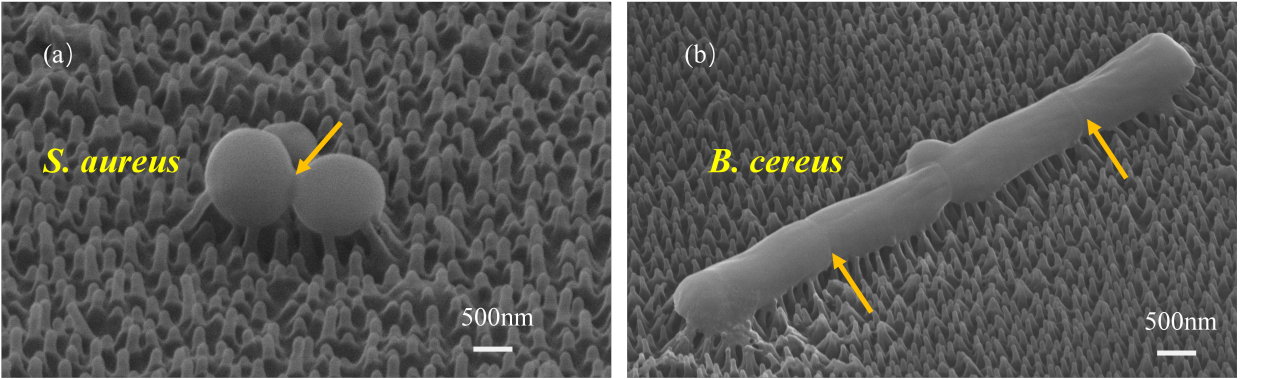


**Fig. S2.** Bacterial cell division occurs on the nanopillar array of cicada wings. (a) *S. areus.* (b) *B. cereus.*
